# Social Skills and Perceived Maternal Acceptance-Rejection in Relation to Depression in Infertile Women

**Published:** 2011-09-23

**Authors:** Fariba Yazdkhasti

**Affiliations:** Department of Psychology, Faculty of Education, University of Isfahan, Isfahan, Iran

**Keywords:** Infertility, Acceptance, Rejection, Social Skills, Depression

## Abstract

**Background:**

This study examines the relationship between infertile women’s social skills and their perception of their own mothers’ acceptance or rejection, and the role this relationship plays in predicting self-reported depression.

**Materials and Methods:**

This was a correlational study. 60 infertile women aged 25 to 35 years participated in a self-evaluation. A Social Skills Inventory, Parental Acceptance and Rejection Questionnaire and Beck Depression Inventory were used to measure social skills, acceptance rejection and depression. Data was analyzed by SPSS software, using independent two-sample t test, logistic regression, and ANOVA.

**Results:**

Findings showed that there are significant differences between depressed and not depressed infertile women in their perceptions of acceptance and rejection by their mothers. Further, women's perceptions of rejection are a more significant predictor of depression among less socially skilled infertile women than among those who are more socially skilled. Less socially skilled women did not show symptoms of depression when they experienced their mothers as accepting. In general the results of this study revealed that poorer social skills were more predictive of depression while good social skills moderate the effect of infertile women’s perceptions of their mothers' rejection. At the same time, the findings showed that infertile women's perceptions of acceptance moderated the effects of poorer social skills in predicting depression.

**Conclusion:**

Results suggest that the perception of mothers’ rejection and poor social skills are the key factors that make infertile women prone to depression.

## Introduction

Infertility is commonly defined by physicians as the inability to conceive after one year of unprotected intercourse (1). Infertility has been characterized as creating chronic stress that can arise due to a variety of psychological difficulties (2, 3). It is sometimes accompanied by crises and emotional tensions such as depression, anxiety and interpersonal problems (2, 3). Greil (4) noted that distinctions between infertile and fertile populations were most pronounced in measures of depression, anxiety, and self-esteem.

Infertile people are more susceptible to depression due to specific factors related to their infertility. It is expected that demographic factors, as a part of social background, are paramount in how an individual meets the problems resulting from infertility (5). It seems that the incidence of psychological disorders in infertile women is much higher, compared to infertile men and their spouses. The psychological consequences of infertility for women are severest (6). It is likely that women's response to infertility is influenced by social and personal factors like parental acceptance or rejection and the women's social skills. Accepting parents are defined as those who show their love or affection toward children physically and verbally. Rejecting parents are defined as those who dislike, disapprove of, or resent their children (7).

Unacceptive parent-child interaction may lead to decreased adjustment in adulthood (7). It means that receiving unacceptive responses from parents (rejective, ambivalent, ambiguous parenting) might lead an individual to appraise the stressor as more threatening, which in turn may have a detrimental impact on adjustment.

Social skills are an individual personality trait that can have profound effects on the nature of interaction with other people as well as on one’s own psychological well-being. Indeed, these two phenomena are theoretically related as the nature of social interactions can affect and be affected by a person’s state of mind and mental health (8). Deficits in social skills have been implicated in depression (8). Social skills are defined as the ability to interact with others in a way that is both appropriate and effective (8). Healthy interpersonal relationships are necessary for healthy psychological development. The inability to present one's authentic self in one’s significant relationships can lead to suppression of self with resulting depression (9).

Many studies have focused on the importance and prevalence of depression in infertility, but very little research has been published concerning preventable predictors of depression among infertile women. The aim of this study was to first identify social (perception of mothers’ acceptance or rejection) and personal (social skills) predictors of depression in infertile women, and then to examine their interaction with depression.

## Materials and Methods

This was a correlational study. The study population included all infertile couples visiting the Isfahan Fertility and Infertility Center and Shahid Beheshti Fertility and Infertility Clinic between April and August 2009. All women had been infertile for four years and above. Follow-up treatment for infertility was at least one year. First, 150 infertile women were selected based on cluster sampling (simple random selection) and then assessed for depression. Informed consent forms were signed by all patients.

The Beck Depression Inventory (BDI), which includes 21 aspects of depression, was created by Beck in 1961 (10). It is a self-report instrument and the reliability (0.96) and validity (0.89) of this test were confirmed during the first decade following its introduction. Scores range as follows: no depression, 0-16; mild depression, 17-27; moderate depression, 28-34; and severe depression, 35-63 (11, 12).

Based on the score in BDI, 30 women with depression and 30 women with no depression were randomly selected. Then these 60 women completed the Social Skills Inventory (13) and Parental Acceptance-Rejection Checklist (mother form) (14).

The Social Skills Inventory is a self-report instrument designed to measure elements of social skills. It was created by Riggio and Canary in 2003 (15). It has six factors and 30 items. It contains statements that assess elements of social skills such as social expressivity, emotional expressivity, social sensitivity, emotional sensitivity, social control, and emotional control.

The Parental Acceptance-Rejection Questionnaire (PARQ) is a self-report instrument which assesses adults’ perceptions of their mothers’ treatment of them when they were about seven through twelve years old. It contains 60 items and two factors. The two factors are acceptance and rejection. It was constructed by Goldberg in 1972 (14). The reliability of the scale was judged as 0.91.

In the current study Cronbach's alpha values for the two instruments were computed. In the Social Skills Inventory, Cronbach's alpha values ranged from 0.68 to 0.89 and in the PARQ they ranged from 0.62 to 0.70. To assess construct validity, the Pearson correlation within each instrument (each subscale and total score) was calculated (16). In the Social Skills Inventory, the correlation between each factor and the total score ranged from 0.34 to 0.90 and in PARQ from 0.88 to 0.97.

To translate PARQ and the Social Skills Inventory into Persian, one bilingual American and one bilingual Iranian worked together in an iterative process from the English instruments. When the Persian instruments were completed, to fit the two scales to Iranian culture, a convenience selected sample of 30 Iranian people and three Iranian psychologists were interviewed. The instruments were then adjusted according to the feedback received in these interviews (Copies of the final instruments are available by request from the author).

## Results

Classification of factors for analysis in PARQ: in the original questionnaire, the factors of this checklist were classified into high and low groups based on the average of each factor, and four combinations were found: (1) acceptance; (2) rejection, (3) ambivalence and (4) avoidance (Table 1). In the present research the above four combinations were also used based on the average score of each factor.

**Table 1:** The four combinations of two factors on the mothers’ report form of the Parental Acceptance and Rejection Questionnaire (PARQ)


Combination name	Factor values	

**Acceptance**	Acceptance	High
	Rejection	Low
**Rejection**	Acceptance	High
	Rejection	Low
**Avoidant**	Acceptance	Low
	Rejection	Low
**Ambivalent**	Acceptance	High
	Rejection	High


**Fig 1:**
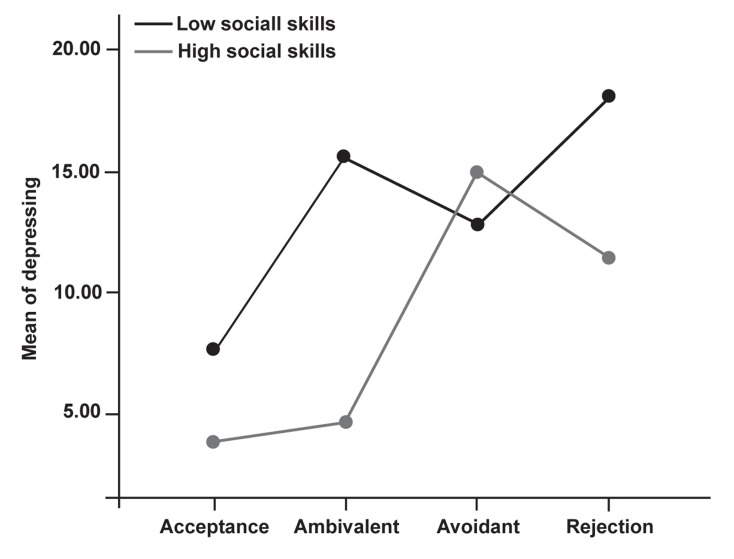
Interaction between social skills and women’s perceptions of mother’s acceptance-rejection

### Logistic regression

Logistic regression was used to determine the relationships between social skills and perception of mothers’ acceptance-rejection variables, and depression. Results showed that the relationships between social skills and depression were significant. Depression was more frequent among individuals with poorer social skills. Also the relationship between depression and perception of mothers’ acceptance-rejection were significant. Thus the rate of depression was significantly higher among individuals experiencing maternal rejection (Table 2).

**Table 2:** Logistic regression to determine the relationships between social skills and perception of mothers’ acceptance-rejection variables and depression


Variables	F	Beta	B	t	r	p

**Mother’s acceptance-rejection**	4.5	0.35	0.07	2.13	0.35	0.04
**Social skills**	35/8	-0.61	-0.49	-5/9	0.61	0.00


### Independent two-sample t tests

The independent two-sample t-tests were used to compare infertile women’s perceptions of their mothers’ acceptance or rejection with the rates of depression and no depression. Results revealed a significant difference between the perception of mothers’ acceptance-rejection among women with depression and with no depression. Rejection was more frequent among women with depression (Table 3).

**Table 3:** The independent two-sample t-tests to compare the perception of mothers’ acceptance-rejection and social skills of infertile women with depression and with no depression


Variables	Two groups	SD Mean	t	Sig.(2-tailed)

**Mother’s acceptance-rejection**	women with depression	28.8 ± 157.9	7.3	0.00
	women with no depression	30.8 ± 101.0		
**Social skills**	women with depression	6.2 ± 77.1	8.4	0.00
	women with no depression	7.08 ± 91.6		


The independent two-sample t-tests were also used to compare infertile women’s social skills with and without depression. Results delineated significant differences between the social skills of infertile women with depression and without depression. Thus the social skills of women without depression were significantly better than those of women who suffered from depression (Table 3).

Among the four combinations of the perception of mothers’ acceptance-rejection (acceptance, rejection, avoidance, and ambivalence) the relationships between ambivalence and no depression, r= 0.55; p<0.05, and acceptance and no depression, r = 0.53; p<0.05 were significant. Among the four factors of social skills, the relationships of three factors, social expressivity, emotional sensitivity and social control, with depression were significant; r = 0.53; p<0.00, r = 0.46; p<0.00, r = 0.67; p<0.00, respectively. The rate of depression was significantly lower among individuals with higher social expressivity, emotional sensitivity, and social control.

### Analysis of variance (ANOVA)

To examine the impact of women’s perception of their mothers’ acceptance-rejection and their social skills on depression, a 2 [social skills (high-low)] × 4 [women’s perception of their mothers’ acceptance- rejection (acceptance, rejection, avoidance, ambivalence)] analysis of variance was conducted. Results of ANOVA revealed a significant interaction between social skills and women’s perception of their mothers’ acceptance-rejection with depression; F (3) = 3.2, p<0.05.

Results of the Tukey HSD indicated that women’s perceptions of rejection and ambivalence are a greater predictor of depression among women with poorer social skills than were the factors of avoidance and acceptance. Among women with good social skills, none of the women’s perceptions of parental acceptance- rejection were predictors of depression (Fig 1).

To examine the impact of women’s perception of their mothers’ acceptance-rejection and their emotional sensitivity (one of the factors of social skills) on depression, a 2 [emotional sensitivity (highlow)] × 4 [women's perceptions of their mothers’ acceptance-rejection (acceptance, rejection, avoidance, ambivalence)] analysis of variance was conducted.

**Fig 2:**
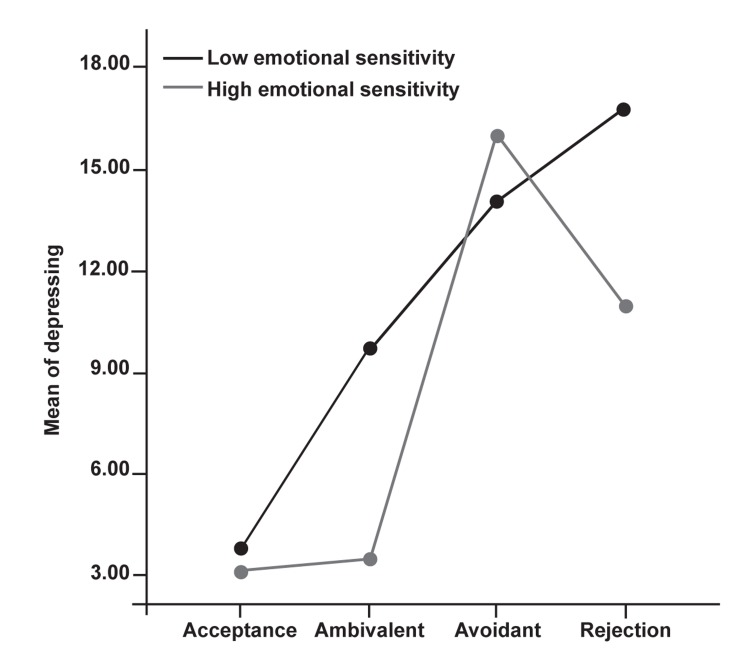
Interaction between emotional sensivity and women’s perceptions of mother’s acceptance-rejection

Results of the ANOVA revealed a significant relation of emotional sensitivity and women’s perception of their mothers’ acceptance-rejection to depression; F (3) = 2.7, p<0.05. Results of the Tukey HSD indicated that women's perceptions of rejection and ambivalence were a greater predictor of depression among less emotionally sensitive women than among the highly emotionally sensitive group. Among women with high emotional sensitivity none of the women's perceptions of their mothers’ acceptance-rejection were a predictor of depression (Fig 2).

To examine the impact of women’s perception of their mothers’ acceptance-rejection and social control (one of the factors of social skills) on depression, a 2 [social control (high- low)] × 4 [women's perceptions of their mothers’ acceptance-rejection (acceptance, rejection, avoidance, ambivalence)] analysis of variance was conducted.

**Fig 3:**
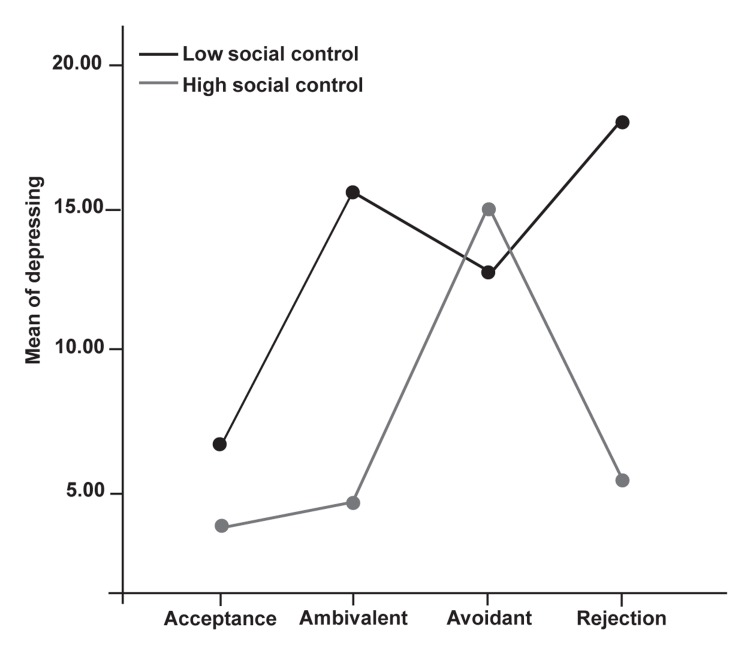
Interaction between social control and women’s perceptions of mother’s acceptance-rejection

Results of the ANOVA revealed a significant interaction of women’s social control and perception of their mothers’ acceptance-rejection with depression; F (3) = 2.8, p<0.05. Results of the Tukey HSD indicated that women’s perceptions of rejection and ambivalence were a greater predictor of depression among less socially controlled women than among the highly socially controlled group. Among women with high social control none of the women's perceptions of their mothers’ acceptance-rejection were a predictor of depression (Fig 3).

## Discussion

These data suggest that perception of mothers’ rejection, as well as poor social skills, are the key factors which make infertile women prone to depression.

According to Schmidt et al. (17) the experience of infertility is shaped by a variety of social interaction. Talking to others can be an important coping strategy for infertile women. Infertility-specific unsupportive interactions would be significantly associated with increased depression symptoms (18, 19). Thus, distancing and unsupportive social interaction, which reflects behavioral and emotional disengagement of the infertile women, appears to be a strong predictor of depressive symptoms and overall psychological distress. This rejection (distancing unsupportive social interaction) may intensify the sense of stigma (disqualification from full social acceptance) associated with infertility and the corresponding psychological sequelae (20). Research indicates that regardless of infertile women’s social skills and their perceptions of their mothers’ acceptance-rejection, it is unclear why some women experience a more powerful sense of stigma from being infertile than other women and why others experience more depression as a result of this stigma. However, the results of the present study show that infertile women had differential resources for depression based on social skills and their perceptions of their mothers’ acceptance or rejection.

## Conclusion

The finding of this study revealed the effect of women’s perception of their mothers’ acceptance as a moderator, and mothers’ rejection and ambivalence as an aggravator of poorer social skills in predicting depression in infertile women. The results of this study also indicated a moderating effect of infertile women’s good social skills on the rejection and ambivalence of their mothers in leading to depression. These women showed fewer symptoms of depression than those with poorer social skills when experiencing their mothers’ rejection or ambivalence. Further research should be performed to corroborate these findings in other Iranian populations, preferably using a national sample.

## References

[1] Mosher WD, Pratt WF (1990). Facundity and infertility in the united states. advance data.

[2] Schneid Kofman N, Sheiner E (2005). Does stress effect male infertility?. A - - debate. Med Sci Monit.

[3] Cox SJ, Glazebrook C, Sheard C, Ndukwe G, Oates M (2006). Maternal self-esteem after successful treatment for infertility. Fertil Steril.

[4] Greil AL (1997). Infertility and psychological distress: a critical review of the literature. Soc Sci Med.

[5] Ghaemi Z, Forouhaui S (2010). Psychological aspect if infertility. International Journal of Fertility and Sterility.

[6] Karamizadeh M, Salsabili N (2010). Survey the psychological disorder of infertility in infertile couples (Couples who under going for ART protocol).International Journal of Fertility and Sterility. International Journal of Fertility and Sterility.

[7] Rohner R P (2007). Handbook for the study of parental acceptance and rejection.USA: Rohner research publications.

[8] Segrin Ch, Taylor M (2007). Positive interpersonal relationships mediate the association between social skills and psychological well-being. Personality and Individual Differences.

[9] Olshansky E, Sereika S (2005). The transition from pregnancy to postpartum in previously infertile women: a focus on depression. Arch Psychiatr Nurs.

[10] Beck AT, Steer RA, Garbin MG (1988). Psychometric properties of the Beck Depression Inventory: twenty-five years of evaluation. Clinical Psychology Review.

[11] Rooshan R (2002). The surveying of comparison the rate of prevalence depression and anxiety between shahed and non shahed students of university.Presented for the Ph.D., Tehran.Tarbeyat Modares University.

[12] Karam sobhani R (1991). The surveying of comparison the rate of prevalence depression in Isfahan.Presented for the Ph.D., Tehran.Tarbeyat Modares University.

[13] Riggio RE (1986). Assessment of basic social skills. Journal of Personality and Social Psychology.

[14] Goldberg LR (1972). Parametrs of personality inventory construction and utilization: Acomparison of predictive strategies and tectics. Multivariate Behavioral Research Monograph.

[15] Riggio RE, Canary DR (2003). Social skills inventory manual.

[16] Cohen RJ, Swerdlik ME (2010). Psychological testing and assessment: an introduction to tests & measurement.

[17] Schmidt L, Thomsen T, Boivin J, Nyboe Anderson N (2005). Evaluation of a communication and stress management training programme for infertile couples. Patient Educ Couns.

[18] Mann SL, Glassman M (2000). Perceived control,coping efficacy, and avoidance coping as mediators between spouses' unsupportive behaviors and cancers patopnts' psychological distress. Health Psychol.

[19] Song YS, Ingram KM (2002). Unsupportive social interactions, availability of social support, and coping: Their relationship to mood disturbance among Aferican Americans living with HIV. Journal of Social and Personal Relationship.

[20] Mindes EJ, Ingram KM, Kliewer W, James CA (2003). Longitudinal analysies of the relationship between unsupportive social interactions and psychological adjustment among women with fertility problems. Social Science & Medicine.

